# Evidence of mitochondrial DNA in the chloroplast genome of *Convallaria keiskei* and its subsequent evolution in the Asparagales

**DOI:** 10.1038/s41598-019-41377-w

**Published:** 2019-03-22

**Authors:** Gurusamy Raman, Seongjun Park, Eun Mi Lee, SeonJoo Park

**Affiliations:** Department of Life Sciences, Yeungnam University, Gyeongsan, Gyeongsan-buk, Republic of Korea

## Abstract

DNA transfer between internal organelles such as the nucleus, mitochondrion, and plastid is a well-known phenomenon in plant evolution, and DNA transfer from the plastid and mitochondrion to the nucleus, from the plastid to the mitochondrion, and from the nucleus to the mitochondrion has been well-documented in angiosperms. However, evidence of the transfer of mitochondrial DNA (mtDNA) to the plastid has only been found in three dicotyledons and one monocotyledon. In the present study, we characterised and analysed two chloroplast (cp) genome sequences of *Convallaria keiskei* and *Liriope spicata*, and found that *C*. *keiskei* has the largest cp genome (162,109 bp) in the Asparagaceae. Interestingly, *C*. *keiskei* had a ~3.3-kb segment of mtDNA in its cp genome and showed similarity with the mt gene *rpl10* as a pseudogene. Further analyses revealed that mtDNA transfer only occurred in *C*. *keiskei* in the Nolinoideae, which diverged very recently (7.68 million years ago (mya); 95% highest posterior density (HPD): 14.55–2.97 mya). These findings indicate that the *C*. *keiskei* cp genome is unique amongst monocotyledon land plants, but further work is necessary to understand the direction and mechanism involved in the uptake of mtDNA by the plastid genome of *C*. *keiskei*.

## Introduction

The engulfment of bacterial endosymbionts led to the development of eukaryotic cells, and the consequent gradual conversion of those bacteria into eukaryotic organelles such as the mitochondrion and chloroplast (cp)^1,2^. During this process, there was a significant transfer of cp and mitochondrial (mt) genes from the endosymbiont genomes into the nuclear genome of the host cell^3^. Lateral and horizontal genetic material transfer between organisms and intracellular gene transfer (IGT) between genomes within organisms are common processes in both prokaryotes and eukaryotes rather than by vertical transfer through sexual reproduction^4–8^. In plants, the intracellular transfer of genetic material between the cp, mt and nuclear genomes is a common process. The IGT of plastid DNA to the mt and nuclear genomes and the transfer of plastid and mtDNA to the nuclear genome are well-documented and regular phenomena in the land plants^4–8^. Also, previous studies reported that nuclear DNA has been transferred to mt genomes in the Fabaceae and Cucurbitaceae due to the presence of a permeable transition pore complex in the mitochondria^9,10^. Nevertheless, land plant plastomes are highly conserved and considered essentially immune to IGT, and it is thought that plant cp genomes do not accept the incorporation of foreign DNA because of the integrity of the plastid membrane^7,8,11^. However, four studies have documented mt gene transfer to plastomes. In angiosperms, the mt pseudogene *cox1* has been transferred into the plastome of *Daucus carota*^11–13^, *rpl2* was transferred in the common milkweed^14^, a complete copy of *ccmB* was transferred in *Anacardium occidentale*^15^, and intergenic sequences were transferred in herbaceous bamboos^16^. In contrast, no evidence of nuclear DNA transfer into the plastid has been reported in any land plant.

In the present study, we characterised and analysed the complete cp genome sequences of two monocotyledon angiosperm plants, *Convallaria keiskei* Miq. and *Liriope spicata* (Thunb.) Lour., and conducted comparative genomics of closely related species in the subfamily Nolinoideae and family Asparagaceae, which revealed that *C*. *keiskei* has the largest cp genome in the Asparagaceae. We also analysed cp pseudogenes in the Asparagales in order to understand the evolutionary histories of these genes. Furthermore, we identified the transfer of mtDNA, including the *rpl10* pseudogene, into the plastome of *C*. *keiskei* based on the full genome sequence of the plastome, and confirmed it using gene-specific primers. Additional work confirmed that mtDNA transfer only occurred in the *C*. *keiskei* cp genome of the Nolinoideae. Finally, molecular evolutionary analyses suggested that *C*. *keiskei* recently diverged. To the best of our knowledge, this is the largest mtDNA segment that has been transferred into the cp genome of the monocotyledon *C*. *keiskei*.

## Results and Discussion

### General features

**Figure 1:**
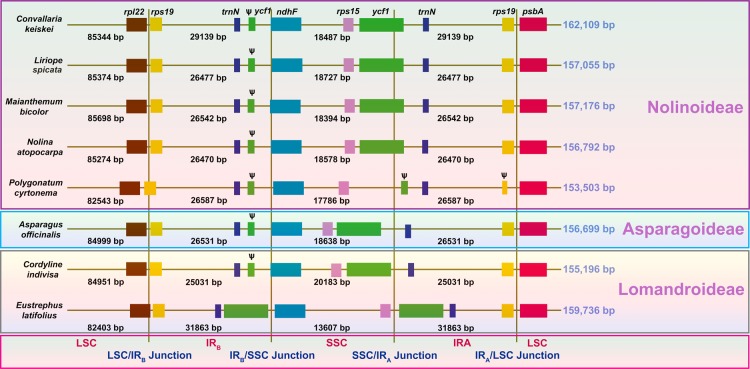
Comparison of the large single-copy (LSC), small single-copy (SSC) and inverted repeat (IR) border regions of Asparagaceae chloroplast genomes. Ѱ indicates a pseudogene. The figure is not drawn to scale.

The plastid genomes of *C*. *keiskei* and *L*. *spicata* are circular molecules that are 162,109 and 157,055 bp in length, respectively. Both cp genomes exhibit a typical quadripartite structure: the large and small single-copy (LSC and SSC, respectively) regions of *C*. *keiskei* are 85,344 and 18,487 bp long, respectively, and there are two parts of an inverted repeat (IR) that are each 29,139 bp long. *L*. *spicata* has a 85,374 bp LSC, a 18,727 bp SSC and a 26,477 bp IR (Supplementary Fig. S1). A total of 136 genes were predicted in both genomes, 115 of which were unique and included 81 protein-coding genes, 30 transfer RNA (tRNA) genes, and 4 ribosomal RNA (rRNA) genes (Supplementary Table S1). Nine protein-coding, eight tRNA, and four rRNA genes were duplicated in the IR regions; however, only a part of the *ycf1* gene was duplicated in the junction of the SSC and IR regions. A total of 17 intron-containing genes (12 protein-coding genes and 5 tRNAs) were present in both cp genomes. The predicted genes were divided into four categories based on their functions. The first category contained 34 genes, including rRNA and tRNA genes; the second category contained 48 genes that were associated with photosynthesis, including subunits of photosystem I and II, photosynthetic electron-transport-chain-component genes, the rubisco large subunit gene, and presumed NAD(P)H dehydrogenase subunit genes; the third category contained genes that were associated with transcription and translation; and the fourth category contained genes related to amino acids, fatty acids, other biosynthesis-related genes, and some genes with unknown functions (Supplementary Table S2). Although the presence and order of the genes in both genomes were similar to those in other Asparagales species, cp genome size in the Nolinoideae varied from 153 to 162 kb (Fig. 1). The average genome size of the Asparagaceae was approximately 157 kb, but the *Polygonatum cyrtonema* cp genome was only 153.5 kb long. The cp genome size of *C*. *keiskei* was 162 kb, indicating that cp genome size is not highly conserved in the Nolinoideae. *C*. *keiskei* has the largest plastome in the Asparagaceae, and the third-largest in the Asparagales behind species in the family Orchidaceae, *Cypripedium formosanum* and *Cypripedium japonicum*.

### Comparative analysis

**Figure 2:**
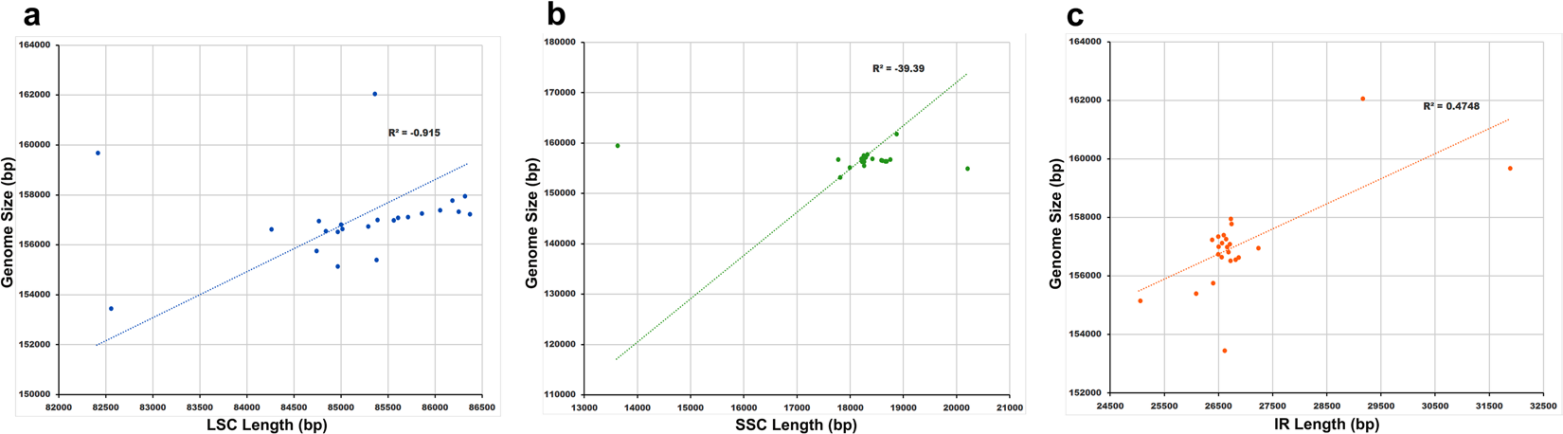
Relationships between Asparagales chloroplast genome sizes and large single-copy (LSC), small single-copy (SSC) and inverted repeat (IR) region lengths.

The LSC/IRB/SSC/IRA boundary regions of the Nolinoideae and those of the closely related subfamilies Asparagoideae and Lomandroideae were compared. Genes in the boundary regions were highly conserved, with small variations in the cp genomes of the Nolinoideae, except for *P*. *cyrtonema*. The LSC region in *P*. *cyrtonema* and *Eustrephus latifolius* is ~82 kb long^17^. The reduction of ~3 kb was caused by the deletion of nucleotides in the intergenic regions of the LSC. Interestingly, the plastomes of the Lomandroideae varied with SSC and IR region size. The ~2 kb SSC region in the *Cordyline indivisa* plastome increased due to a complete shift of *ycf1* from the IR region. However, in *E*. *latifolius*, this was only ~13.6 kb long because *ycf1* was in the IR regions, which increased the IR size to ~31.6 kb. The IR region in *Convallaria keiskei* increased by ~3 kb due to the insertion of mtDNA into its plastome (Fig. 1). Sizes of the LSC, SSC and IR regions were analysed in 23 Asparagaceae cp genomes in order to understand the diversity of the family (Fig. 2). Typical sizes of the LSC, SSC and IR regions in most of the Asparagaceae were ~85, ~18 and ~26 kb, respectively. The size of the LSC region varied from 82,403 to 86,356 bp (Fig. 2a) because of the presence of indels. The SSC region varied from 13,607 to 20,183 bp in length (Fig. 2b) and the IR regions varied from 25,031 to 31,863 bp in length (Fig. 2c), indicating the presence of an indel of the *ycf1* gene in both the SSC and IR regions in the plastomes of the Asparagaceae. When compared with another subfamily of the Asparagaceae, the sizes of the LSC, SSC and IR regions of the Lomandroideae and Nolinoideae were extremely diverse, which reflects genome size variation and suggests that these plastomes are not highly conserved. There was a close association between LSC length and genome size, whereas associations between SSC and IR lengths and genome size were highly variable.

### Comparative analysis of the pseudogene *infA* with other Asparagales cp genomes

All of the photosynthetic- and transcriptional-related genes were present in the plastomes of both species, as in other Asparagales. However, some differences were observed in the protein-coding genes of the Asparagales. mVISTA was used to study sequence variations in the Nolinoideae subfamily and other Asparagales plastomes, which revealed that both coding and non-coding regions are not highly conserved in Asparagales plastomes (Supplementary Fig. S2). Specifically, large differences were found in the protein-coding and intergenic regions of the LSC in the plastomes of the Asparagales that contained a large number of pseudogenes, intron deletions and inversions (Supplementary Table S3). Most of these genes were related to transcription and translation, and are essential for land plants. The pseudogenes included *accD*, *infA*, *rpl23*, *rpl32*, *rps2*, *rps16*, *rps19*, and *ycf1*. The coding regions in the Nolinoideae plastomes of *C*. *keiskei*, *L*. *spicata*, *Maianthemum bicolor*, and *Nolina atopocarpa* were highly conserved, but differed to those of *P*. *cyrtonema*. The protein-coding regions of the Nolinoideae were extracted and evaluated in order to identify divergent hotspots in the coding regions (Supplementary Fig. S3). Most of the protein-coding genes were highly conserved; however, minor divergences were detected. The protein-coding genes *accD*, *cemA*, *ccsA*, *matK*, *ndhF*, *rpl22*, *rpl32*, and *rps15* had slightly diverged in the Nolinoideae because of an indel in their LSC, IR and SSC regions. The *infA* gene had highly diverged due to the presence of a pseudogene in the *Convallaria*, *Liriope* and *Nolina*^18^ cp genomes.

**Figure 3:**
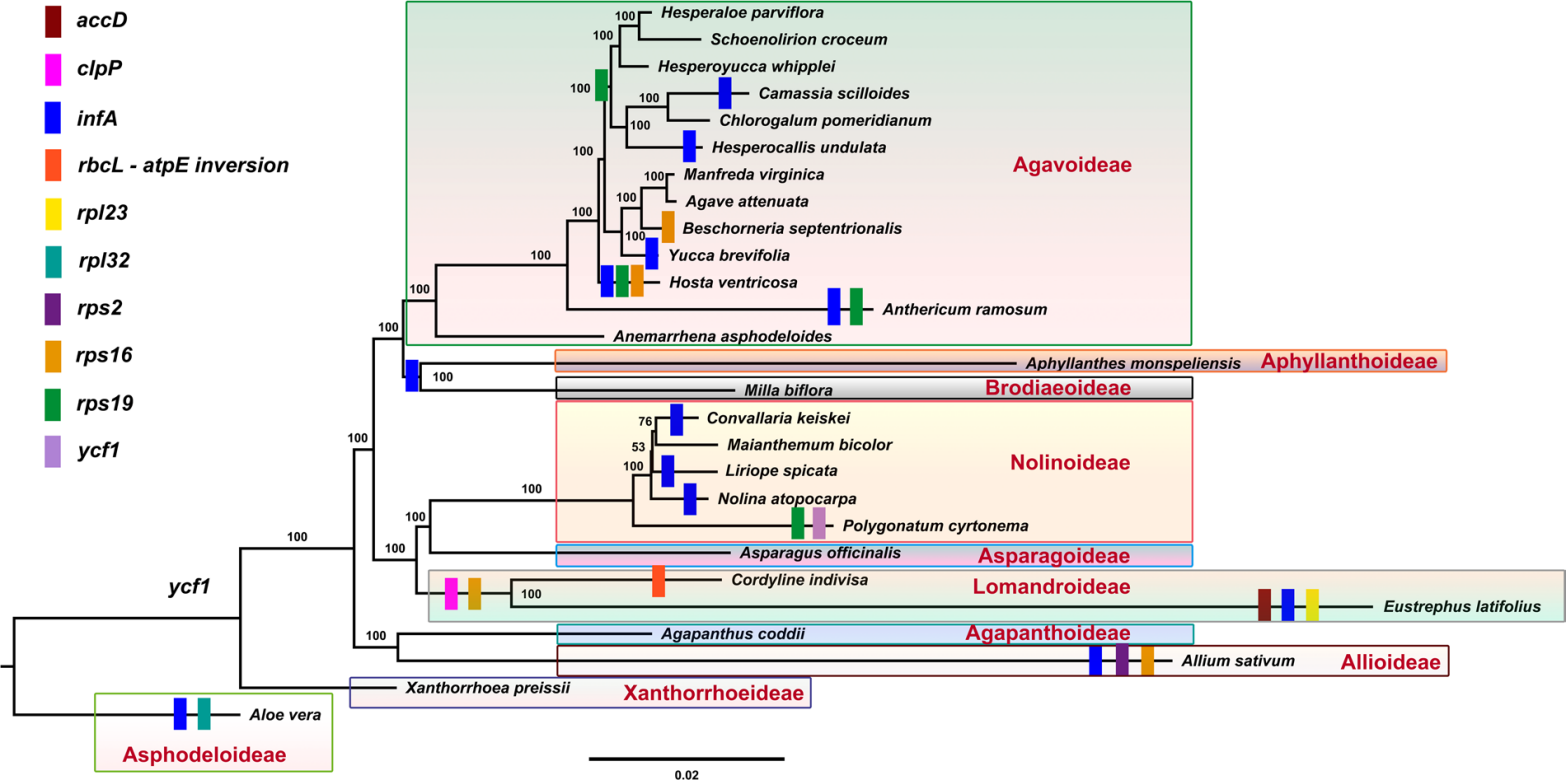
Molecular phylogenetic tree of 27 Asparagales taxa based on 68 protein-coding genes in the chloroplast genome. The tree was constructed by maximum likelihood analysis of the conserved regions using the RAxML program and the GTR + G + I nucleotide model. The stability of each tree node was tested by bootstrap analysis with 1,000 replicates. Bootstrap values are indicated on the branches, and the branch length reflects the estimated number of substitutions per 1,000 sites. *Aloe vera* and *Xanthorrhoea preissii* were set as the outgroups.

The cp gene *infA* of *C*. *keiskei* and *L*. *spicata* was compared with those in other Asparagales and was found to be a pseudogene in both the *C*. *keiskei* and *L*. *spicata* cp genomes, whereas in the plastome genomes of *M*. *bicolor* and *P*. *cyrtonema* it was a functional *infA* gene. The functional *infA* gene sequence was highly variable in the Nolinoideae. The *infA* gene on the *L*. *spicata* cp genome was only 87 bp long, possibly due to a frameshift mutation caused by the insertion of 4 bp at a position between 59 and 62 bp. Two base pairs had been deleted at 183–184 bp in the *infA* gene of *C*. *keiskei*, resulting in it becoming a pseudogene with a total size of 186 bp. A similar pattern was observed in *N*. *atopocarpa*, in which 8 bp had been deleted in the *infA* gene that became a pseudogene with a total size of 72 bp. Phylogenetic analyses revealed that the loss of *infA* occurred independently in the Nolinoideae (Fig. 3). Previous studies have reported that the *infA* gene was independently lost from other monocotyledons, such as most of the Agavoideae, Allioideae, Aphyllanthoideae, Asphodeloideae, Brodiaeoideae, Lemnoideae, Lomandroideae, and other angiosperm lineages^17–21^. A comparison of the other plastome protein-coding genes of the Asparagales revealed that *accD*, *rpl23*, *rpl32*, *rps2*, *rps16*, *rps19* and *ycf1* are pseudogenes. Comparative analysis revealed that all of the pseudogene or gene loss occurred independently in the Asparagales, except for *rps16* in the Lomandroideae (Fig. 3). However, the cp genomes of several species of the Lomandroideae should be investigated in order to elucidate the evolution of *rps16*. Although several cp genes were deleted during evolution, these deletions are not related to the taxonomy of the Asparagales. Taken together, these results suggest that most cp gene loss occurred independently across the Asparagales, as well as the angiosperms.

### Synonymous and non-synonymous substitutions

**Figure 4:**
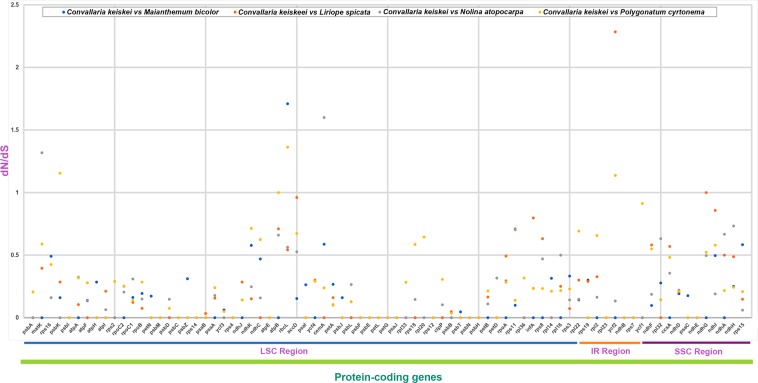
Ratio of non-synonymous (dN) to synonymous (dS) substitutions of 68 protein-coding genes in the Nolinoideae. Blue dots indicate the dN/dS ratio of *Convallaria keiskei* vs *Maianthemum bicolor*, orange dots indicate *C*. *keiskei* vs *Liriope spicata*, grey dots indicate *C*. *keiskei* vs *Nolina atopocarpa*, and yellow dots indicate *C*. *keiskei* vs *Polygonatum cyrtonema*.

In genome evolution studies, the ratio of non-synonymous (dN) to synonymous (dS) substitutions is an important indicator^22^. We calculated the dN/dS ratio in the Nolinoideae using *C*. *keiskei* as a reference genome. The majority of genes in the Nolinoideae genomes had ratios of less than 1.0, with the exceptions of *matK*, *psbK*, *rbcL*, *cemA*, and *ycf2*. *ycf2* had the highest ratio (~2.28), followed by *rbcL* (1.7), *cemA* (1.6), *matK* (1.3) and *psbK* (1.15), indicating that these genes are not conserved in the cp genome (Fig. 4). *cemA*, *rbcL* and *psbK* are involved in photosynthesis, *matK* encodes maturase and the function of *ycf2* is unknown. Although a missense mutation occurred in these genes, they are under positive selection in Nolinoideae cp genomes, possibly by adapting to changing ecological conditions. One-third of plastid genes, including self-replication- and photosynthesis-related genes, evolved under positive selection in the Poaceae^23^. In contrast, in the Nolinoideae, the substitution ratios were less than one for most of the photosynthetic-related genes, except for *cemA*, *rbcL* and *psbK*, which are transcription and translation genes that are more highly conserved than other genes in cp genomes because of strong functional constraints.

### Codon usage

**Figure 5:**
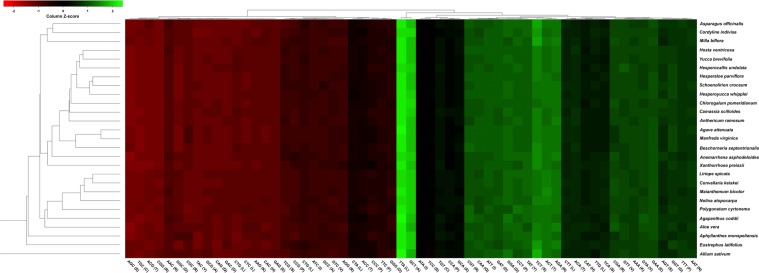
Codon distributions of chloroplast protein-coding genes in the Asparagales. Green indicates a high relative synonymous codon usage (RSCU) value and red indicates a low RSCU value. Hierarchical clustering (average linkage method) was performed for the codon patterns (x-axis).

Basal eudicots encode AUG as the initiation codon for most protein-coding genes, but the *Convallaria* and *Liriope* cp genomes encode an alternative starting codon (ACG) for *rpl2*. A similar type of codon was observed in all of the Asparagales cp genomes. An analysis of the codon usage patterns of 68 unique cp protein-coding genes in 27 Asparagales taxa revealed that 294,399 codons were present in the protein-coding genes. Figure 5 is a heatmap of codon usage in the Asparagales. A relative synonymous codon usage (RSCU) value of <1 (red colour) indicates weak codon bias, and a value of >1 (green colour) denotes strong codon bias. Figure 5 shows that the half of the codons (28) that ended with G/C (denoted in red) are not frequently used in the Asparagales, whereas the 31 codons that ended with A/T (denoted in green) and had high RSCU values are used by all species of Asparagales. Among these, the codons TTA and GCT had high RSCU values of 2.009791 and 1.897735, respectively. Similar results have been obtained in many other land plant and algal lineages^24^. The high RSCU values of the codons indicate amino acid functions or peptide structures that inhibit transcriptional errors in cp genomes.

### Phylogenetic analysis

To elucidate the phylogenetic relationships of the Asparagales, 68 cp protein-coding genes shared by 27 genomes were investigated. The phylogenetic tree was divided into three groups: the Xanthorrhoeaceae, Amaryllidaceae, and Asparagaceae (Fig. 3). The Xanthorrhoeaceae is basal to the rest of the Asparagales. Two major clades formed in the Asparagaceae, with the Lomandroideae, Asparagoideae and Nolinoideae in one clade and the Agavoideae, Aphyllanthoideae and Brodiaeoideae in the other. The Nolinoideae is a sister group to the Asparagoideae, with a 100% bootstrap (BS) value. In the Nolinoideae, *Polygonatum* is the basal group with a 100% BS value, and *L*. *spicata* is a sister species to *M*. *bicolor* and *C*. *keiskei* with a very weak 53% BS value, although the BS value of *M*. *bicolor* and *C*. *keiskei* was 76%. This weak BS value may have been caused by indels and nucleotide differences in the protein-coding genes of their respective cp genomes.

### Divergence time estimation

**Figure 6:**
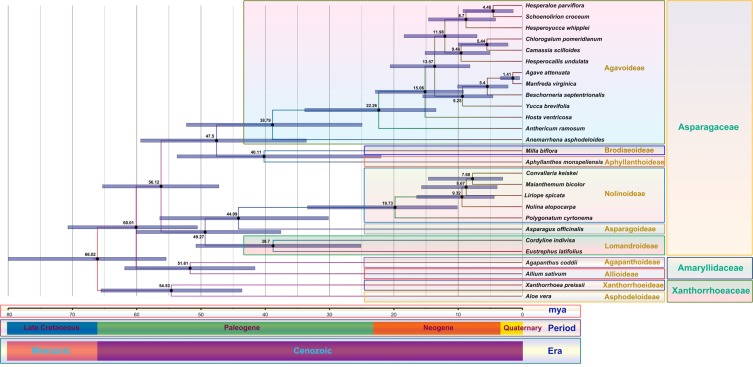
Molecular clock based on 68 protein-coding genes of 27 Asparagales chloroplast genomes using BEAST. Mean age estimates (in millions of years) are shown along the branches. Grey bars represent the 95% posterior density credibility intervals for node ages.

The aim was to estimate divergence time for the Nolinoideae, but due to a lack of calibration points, we included other species of Asparagales. Divergence time was estimated using previous data of the Asparagales, which were similar to those obtained in the present study. In addition, the species used in both the maximum likelihood phylogenetic tree and the divergence analysis were the same. Among the Asparagales, the Xanthorrhoeaceae basal group (*Aloe vera* and *Xanthorrhoea preissii*) diverged 54.35 million years ago (mya) (95% highest posterior density (HPD): 65.48–43.54 mya), followed by the Amaryllidaceae at 51.61 mya (95% HPD: 61.78–41.51 mya) (Fig. 6). In the Asparagaceae, the Asparagoideae (*A*. *officinalis*) is the sister group to the Nolinoideae and diverged at 44.09 mya (95% HPD: 56.33–30.09 mya). Chronogram results from a BEAST analysis revealed that all of the speciation events within Nolinoideae occurred from 56.33 to 2.97 mya. *Polygonatum* diverged from the ancestor of all other members of the Nolinoideae at 19.73 mya (95% HPD: 33.35–10.04 mya), whereas *Liriope* diverged at 8.67 mya (95% HPD: 15.61–3.85 mya). Among the Nolinoideae, *C*. *keiskei* and *M*. *bicolor* diverged over a relatively short amount of time at 7.68 mya (95% HPD: 14.55–2.97 mya). Interestingly, mtDNA transfer only occurred in the cp genome of *C*. *keiskei*, and the pseudogenization of *infA* occurred in all species of the Nolinoideae except for *M*. *bicolor*. Although *M*. *bicolor* is a sister species to *C*. *keiskei*, the *M*. *bicolor* genome was highly conserved during evolution and could have recently diverged. In order to confirm this, more Nolinoideae cp genomes should be investigated.

### Analysis of mtDNA transfer into the *C*. *keiskei* cp genome

**Figure 7:**
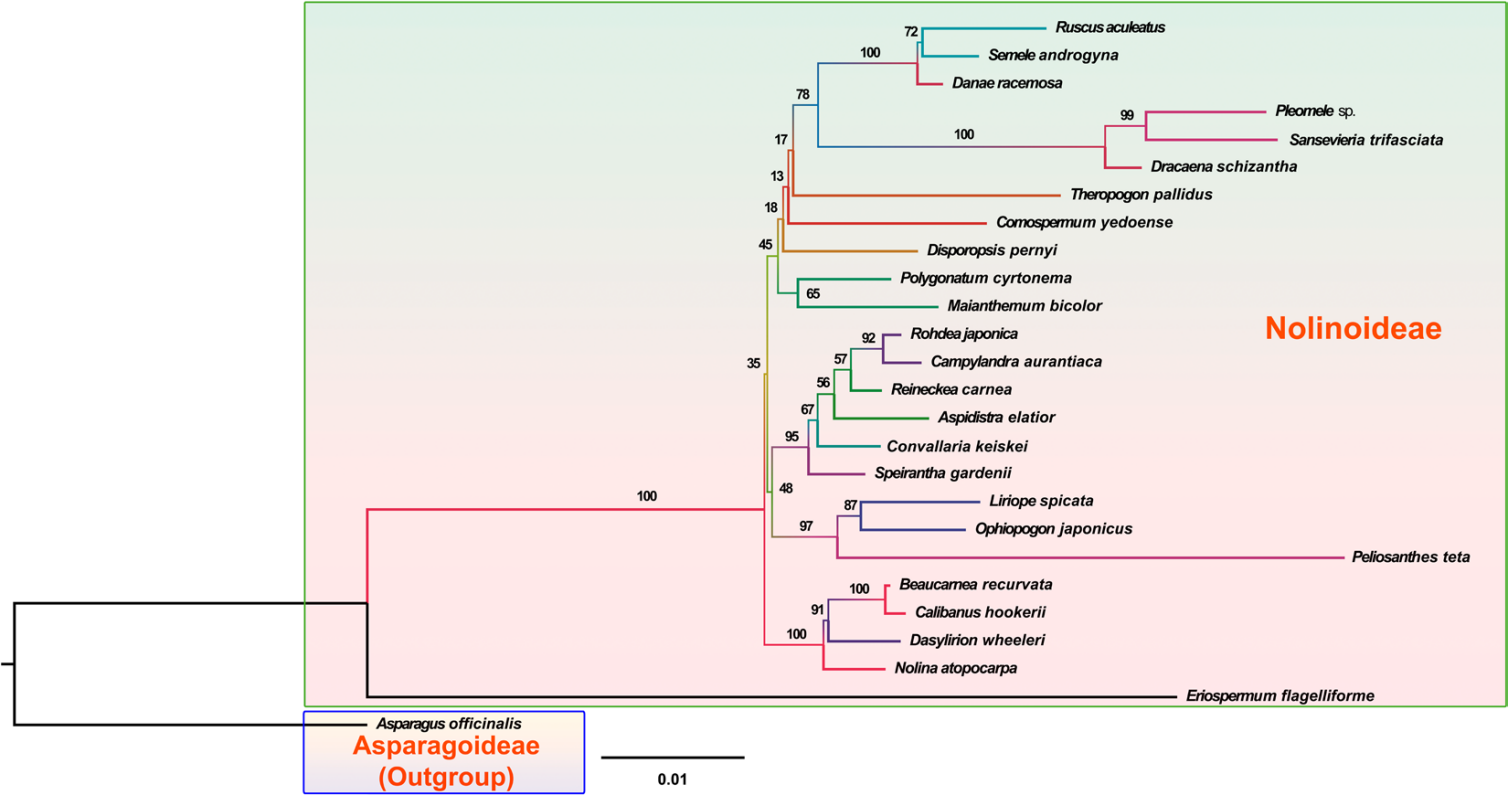
Molecular phylogenetic tree analysis of four chloroplast genes (*atpB*, *matK*, *ndhF* and *rbcL*) in the Nolinoideae subfamily. The tree was constructed by maximum likelihood analysis of the conserved regions using the RAxML program and the GTR + G + I nucleotide model. The stability of each tree node was tested by bootstrap analysis with 1,000 replicates. Bootstrap values are indicated on the branches, and the branch length reflects the estimated number of substitutions per 1,000 sites. *Asparagus officinalis* was set as the outgroup.

**Figure 8:**
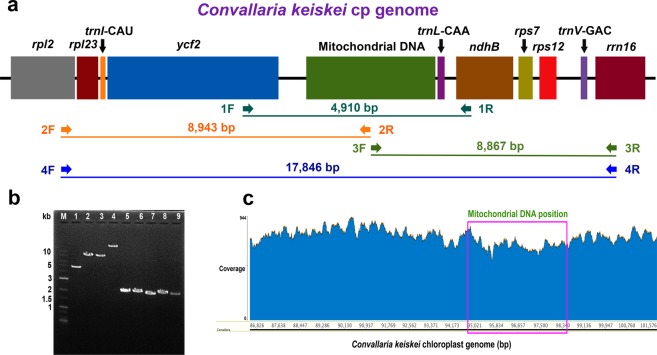
Analysis of mitochondrial DNA transfer in the chloroplast (cp) genome. (**a**) Position of four sets of gene-specific primers in the cp genome of *Convallaria keiskei*. (**b**) Polymerase chain reaction confirmation of the presence or absence of mitochondrial DNA in the Nolinoideae subfamily cp genome using agarose gel electrophoresis. Lane M: SolGent^™^ 1 kb Plus DNA Ladder; Lanes 1–4: *C*. *keiskei* cp genome (Lane 1: *ycf2*–*ndhB*, Lane 2: *rpl2* mitochondrial DNA region, Lane 3: mitochondrial DNA region–*rrn16*, and Lane 4: *rpl2*–*rrn16*); Lane 5: *Reineckea carnea*; Lane 6: *Rohdea japonica*; Lane 7: *Tupistra aurantiaca*; Lane 8: *Speirantha gardenii*; and Lane 9: *Aspidistra* spp. (**c**) Sequencing-depth coverage of the mitochondrial DNA insert region (94,989–98,301 bp) in the *C*. *keiskei* cp genome.

Whole-genome cp sequences are ideal for comparing mtDNA transfer into the cp genome in *C*. *keiskei* with that in other species of the Nolinoideae through phylogenetic analysis, but there is a lack of whole-genome plastome sequence data that cover all Nolinoideae species. Therefore, in the present study, four cp marker genes (*matK*, *rbcL*, *atpB*, and *ndhF*) were used to investigate the relationship between *C*. *keiskei* and other species of the Nolinoideae. The phylogenetic results showed that *Eriospermum flagelliforme* is the basal group in the Nolinoideae, and there were three major clades with weak BS values: *N*. *atopocarpa* formed one clade, *C*. *keiskei* and *L*. *spicata* formed the second, and *M*. *bicolor* and *Polygonatum* formed the third (Fig. 7). However, *C*. *keiskei* and *L*. *spicata* formed sister clades. In order to place the mtDNA transfer event in an evolutionary context, species in the *Convallaria* clade (*Speirantha gardenii*, *Aspidistra elatior*, *Reineckea carnea*, *Camphylandra aurantiaca*, and *Rohdea japonica*) were used to identify whether they shared the mt segment of DNA that is in the *C*. *keiskei* plastome genome. Gene-specific primers were designed to determine whether the mt spacer region in *Convallaria* was similar in size to that in other closely related species of Nolinoideae (Fig. 8a). Polymerase chain reaction (PCR) results confirmed the presence of mtDNA in only the *C*. *keiskei* cp genome, not in other species of Nolinoideae (Fig. 8b). Three further primers were used to confirm the presence of mtDNA in the *C*. *keiskei* cp genome (Fig. 8a), and a PCR confirmed that mtDNA was present in the intergenic regions between *ycf2* and *trnL* (Fig. 8b). In addition, all Illumina sequencing reads were mapped to the *C*. *keiskei* plastome assembly to understand whether the presence of the mt-like insertion in the IR region belongs to the cp genome of *C*. *keiskei*. The analysis showed that the depth coverage of *C*. *keiskeii* plastome is ~428× and the coverage is uniformly distributed across the cp genome regions (Fig. 8c). Also, the depth coverage of mt-like insertion region is very similar to cp genome regions of *C*. *keiskei*. Correspondingly, all Illumina sequencing reads were mapped with mitochondrial genes of *Amborella* (as a reference) to identify the depth coverage of mitochondrial genes of *C*. *keiskei*. The depth coverage of *C*. *keiskei* mitochondrial genes is ~15× (data not shown). Therefore, the analysis confirmed that the presence of the mt-like insertion in the IR region is associated with the cp genome of *C*. *keiskei*.

A total of 3,313 mt-like nucleotide sequences were identified in the *C*. *keiskei* cp genome using BLASTN. These sequences were integrated upstream of tRNA^Leu^ (*trnL*) and downstream of *ycf2* (94,989–98,301 bp in the IRB region) in the plastid genome (Supplementary Fig. S4). The inserted sequences included intergenic regions of the mitogenome, fragments of mt genes, and sequences of unknown providence. Illumina sequencing reads were mapped to the *C*. *keiskei* plastome assembly and showed that it was uniformly distributed across the region and confirmed the presence of a mt-like insertion in the IR region (Fig. 8c). The mt-like sequences were BLASTed against all other genes in the US National Centre for Biotechnology Information (NCBI) database, which revealed that 994 nucleotide sequences had 93.2% similarity with the mt genome of the monocotyledon *Cocos nucifera*. Of the 994 bp, 386 bp were identical to the partial mt *rpl10* gene, which was present as a pseudogene (Supplementary Fig. S5). The remaining 608 bp were in the intergenic regions of *trnL* and *rpl10*. Previous studies have found that IGT in the genome, even if the fragment comprises a gene or genes in higher land plants, usually does not encode any functional property in the recipient genome^4,5,7^, and intracellularly transferred DNA is generally only transiently maintained in the recipient genome over evolutionary time^4,5,7^. Despite this, the presence of a ~2 kb DNA insert sequence in the *C*. *keiskei* cp genome shows that there is no homology with the mt genomes of any other land plants or sequences in GenBank. The lack of mt and nuclear genomic information for *C*. *keiskei* limits our ability to investigate the unknown ~2 kb DNA insert in the *C*. *keiskei* cp genome, but it could have been caused by a unique sequence in the *C*. *keiskei* mt or nuclear genomes.

Cp genome segments ranging from 17 to 130 kb in length have been identified in the mt genomes of many monocotyledons^25–31^ and seed plants^10,26,32–39^, and plastid DNA has been found in the nuclear genomes of *Arabidopsis*, soybean and other species^40,41^. In addition, nuclear DNA has migrated into the mt genomes of *Cucumis melo*^9^, *Arabidopsis*^42^, maize^43^, sugar beet^44^, rice^30^, and wheat^45^, and migration in the opposite direction (mtDNA to the nuclear genome) has also been reported in several angiosperms^46–48^. However, DNA transfer from the mt to the cp has only been identified in four families of land plants. In the dicotyledons *Daucus* and *Cuminum*, 1.5 kb of mtDNA has been found in the *rps12*–*trnV* intergenic spacer of IR regions^11–13^, *Asclepias syriaca* (milkweed) has 2.4 kb of mt-like DNA in the *rps2*–*rpoC2* intergenic spacer of the LSC region^14^, and 6.7 kb of mtDNA has been inserted in *ycf2*–*trnL* in the plastid IR regions of cashew (*A*. *occidentale*) plastomes^15^. Regarding monocotyledons, 2.7 kb of mtDNA has been inserted into the *trnI*-CAU–*trnL*-CAA intergenic spacer in IR regions of the herbaceous bamboo *Pariana*^16^. In *C*. *keiskei*, 3.3 kb of mtDNA is in the intergenic spacer of *ycf2*–*trnL* in plastid IR regions. The location of the mtDNA insertion in the IR region (intergenic region between *ycf2* and *trnL*) of the *C*. *keiskei* plastome is similar to that in the *A*. *occidentale* plastid genome^15^. However, the sizes of the mtDNA insertions and insertion sequences vary among genomes. When comparing the insertion of mtDNA with the other four plastomes, common IGT insertion events occur in the non-coding spacer regions of the plastomes. This integration may be random or is facilitated by homologous recombination; however, any interruption in plastid coding sequences that decreases overall fitness would probably be purged^15^. Among the mtDNA-integrated plastomes of land plants, a high GC content was observed in *C*. *keiskei* (45.7%), followed by *Pariana* (44.7%), *Daucus* (44.0%), *A*. *occidentale* (43.5%) and milkweed (40.3%). In addition, no deletions of plastid sequences were observed in the mtDNA-inserted region in the *C*. *keiskei* plastome, and the same was true of the milkweed and cashew plastid genomes. However, mtDNA insertion was accompanied by the deletion from their plastid genomes of 339 bp in the carrot and 1,379 bp in bamboo. All of these studies reported that mtDNA transfer occurred in common ancestors, such as Apiaceae for *Daucus* and *Cuminum*, Apocynaceae for milkweed, and *Anacardium* and bamboo for *Pariana* and *Eremitis*. However, we found that mtDNA transfer only occurred in the *C*. *keiskei* cp genome, and not in the common ancestor plants of the Nolinoideae. Further studies are required to ascertain why mtDNA insertion is restricted to the *C*. *keiskei* cp genome of the Nolinoideae.

How mtDNA was integrated into the *C*. *keiskei* cp genome is unknown. Because plastids have a double membrane, they are generally unable to take up DNA^4,5,7,8,14^; however, stress, transformation, and double-membrane breach can result in the uptake of foreign DNA. Although gene transfer could occur by IGT, there is no evidence that it plays a role in the IGT mechanism. Further studies are needed to understand intra- and inter-genomic DNA transfer and recombination in the cp genome.

## Conclusion

he *C*. *keiskei* plastome is longer than that of most monocotyledon flowering plants due to an insertion of ~3.3 kb of mtDNA in the IR regions. However, some variations were identified. To the best of our knowledge, this is the first report that *C*. *keiskei* has the largest cp genome in the Asparagaceae, and the second report of mtDNA transfer into monocotyledon plastomes. Analysis of the large amount of angiosperm cp genome sequence data in the NCBI database strongly suggests that this is a rare event in monocotyledons. Taken together, these findings indicate that the *C*. *keiskei* cp genome is unique amongst monocotyledons, and further investigation of the mt genome is required to understand the direction and mechanism of mtDNA uptake by the plastid genome in *C*. *keiskei*.

## Methods

### Genomic DNA extraction and sequencing

Fresh leaf material from *C*. *keiskei* and *L*. *spicata* was collected from the Choijung and Palgong mountains, South Korea, respectively. Total genomic DNA was extracted using a modified cetyl trimethylammonium bromide method^49^. Whole-genome sequencing was performed using an Illumina HiSeq 2500 (Phyzen Ltd., South Korea) and a paired-end (PE) library of 2 × 150 bp and an insert size of ~550 bp. A total of 33,272,066 and 26,965,288 raw reads of *C*. *keiskei* and *L*. *spicata*, respectively, were obtained, and PE Illumina reads were assembled *de novo* using Velvet v1.2.10^50^ with multiple *k*-mers. The initial plastid contigs of both genomes were assembled using Geneious v7.1.8 (Biomatters, New Zealand). The sequencing data and gene annotations of both genomes were submitted to GenBank and assigned accession numbers of MH60946 for *C*. *keiskei* and MH60945 for *L*. *spicata*.

### Cp genome annotation and analysis

The initial annotation of the cp genomes was conducted using the online DOGMA tool^51^. From this initial annotation, putative starts and stops, and intron positions were identified based on comparisons with homologous genes in *N*. *atopocarpa*, *P*. *cyrtonema*, and *Nicotiana tabacum*. The tRNAs identified were confirmed using tRNAscan-SE^52^. A circle map of cp genomes was drawn using the OGDRAW program^53^.

### Comparative genome analysis

The complete cp genome sequences of both *C*. *keiskei* and *L*. *spicata* were compared with 14 other cp genomes in the Asparagales using the mVISTA program in Shuffle-LAGAN mode^54^. The *C*. *keiskei* cp genome was set as a reference. Boundary regions between the LSC, IR and SSC and their lengths were compared and analysed using cp genomes of the Asparagaceae.

### Analysis of dS and dN substitution rates

The *C*. *keiskei* cp genome sequence was compared with those of the Nolinoideae species *L*. *spicata*, *M*. *bicolor*, *N*. *atopocarpa* and *P*. *cyrtonema*. dS and dN substitution rates were analysed by the same individual, and functional protein-coding gene exons were separately extracted and aligned using Geneious v10.2.4. The aligned sequences were translated into protein sequences and analysed by DnaSP^55^.

### Codon usage

Codon usage was determined for all protein-coding genes of Asparagales cp genomes. Codon-usage distributions were visualised in the form of heatmaps of 27 species of Asparagales, and histograms were generated using the Heatmapper program with RSCU values^56^. RSCU values were calculated by counting the number of times a particular codon was observed, relative to all codons for a given amino acid that exhibited similar probabilities^57^. A RSCU value of <1.00 indicated a codon that was used less frequently than expected, and a value of >1.00 indicated a codon that was used more frequently than expected.

### Phylogenetic tree

The jModelTest 2 v0.1.10 program was used to analyse the general GTR + G + I model for protein-coding sequences using optimized parameters^58^. Phylogenetic analyses of four cp genes in the Nolinoideae subfamily (*atpB*, *matK*, *ndhF*, and *rbcL*) and 68 protein-coding genes in 27 Asparagales cp genomes were separately performed using the maximum likelihood method in RAxML v7.2.6 with 1,000 BS replicates^59^.

### PCR amplification of mtDNA insertions in Nolinoideae species

In order to detect mtDNA insertions in the cp genomes of the Nolinoideae, DNA samples of *Aspidistra* spp., *Reineckea carnea* (Andrews) Kunth, *Rohdea japonica* (Thunb.) Roth, *Speirantha gardenii* (Hook.) Baill. and *Tupistra aurantiaca* (Baker) Wall ex Hook.f. were obtained from the DNA Bank of the Royal Botanic Gardens, Kew (http://data.kew.org/dnabank/DnaBankForm.html) and used as templates, and gene-specific primers that were designed based on the plastid genes between *ycf2* and *trnL* (1F and 1R) were used. Further, to confirm the presence of mtDNA in the *C*. *keiskei* cp genome, three different primer sets were designed (Fig. 8a; Supplementary Table S4). The first primer was between *rpl2* and the mtDNA region (2F and 2R), the second was between the mtDNA region and *rrn16* (3F and 3R), and the third was between *rpl2* and *rrn16* (4F and 4R). All of these primers were designed using Primer3 in Geneious v7.1.8. Long PCR products were amplified using gene-specific primers, and the PCR mixture (25 μL) contained 10 pM of primer, 250 ng of genomic DNA, 1X PrimeSTAR GXL buffer with 1 mM MgCl_2_, 0.2 mM of each deoxynucleotide triphosphate (dNTP), and 0.625 U/μL of PrimeSTAR GXL DNA Polymerase (Takara Bio Inc., CA, USA). A two-step PCR amplification was performed in an Arktik thermal cycler (Thermo Fisher Scientific, MA, USA) programmed with an initial denaturation at 98 °C for 2 min, 30 cycles at 98 °C for 10 s, 68 °C for 10 min and 68 °C for 10 min. The 2-μL PCR product was separated using 1.5% agarose gel stained with ethidium bromide staining solution in 1X tris acetate ethylenediaminetetraacetic acid. The image of the gel was digitized using the UVITEC Cambridge system (Cleaver Scientific Ltd., Warwickshire, UK).

### Molecular evolution tree

A total of 68 protein-coding genes from 27 Asparagales cp genomes were used for the molecular divergence analyses. A molecular clock tree was constructed using BEAST v2.1 (Centre for Computational Evolution, University of Auckland, New Zealand)^60^. A relaxed clock log-normal model was implemented using Markov Chain Monte Carlo chains that were run for 300 million generations with a 10% burn-in and were sampled every 1,000 generations. A GTR nucleotide substitution model was used with a gamma distribution and four rate categories. A Yule tree prior was used to estimate divergence times and creditability intervals. The sample size was evaluated using Tracer v1.6 analysis software (Institute of Evolutionary Biology, University of Edinburgh, Edinburgh, UK)^61^, and tree data were summarised using TreeAnnotator v2.1.2 (Centre for Computational Evolution, University of Auckland, New Zealand)^60^. Multiple calibration points were set for the divergence of the Amaryllidaceae at 51.2 ± 6.0 mya (42–61.8 mya), for the divergence of the Asparagaceae at 56.4 ± 5.3 mya (48.1–65.5 mya), and for the divergence of the Xanthorrhoeaceae at 55.6 ± 5.5 mya (48–66.0 mya)^62^, and implemented with a log-normal distribution.

## Supplementary information

Supplementary files
