# Supplementary material for: Evidence of mitochondrial DNA in the chloroplast genome of Convallaria keiskei and its subsequent evolution in the Asparagales
Source: Sci Rep. 2019 Mar 22;9:5028. doi: 10.1038/s41598-019-41377-w (PMC6430787; doi:10.1038/s41598-019-41377-w)
Supplement: Supplementary file 1 — Supplementary files [file 41598_2019_41377_MOESM1_ESM.pdf]

Evidence of mitochondrial DNA in the chloroplast genome of *Convallaria keiskei* and its subsequent evolution in the Asparagales

G. Raman, S. Park, E. M. Lee & S. J. Park*

Department of Life Sciences, Yeungnam University, Gyeongsan, Gyeongsan-buk, Republic of Korea

*Corresponding author: S. J. Park, Department of Life Sciences, Yeungnam University, Gyeongsan, Gyeongsan-buk, Republic of Korea 38541; Tel.: +82 (53) 810-2377; Fax: +82 (53) 810-4618; Email: sjpark01@ynu.ac.kr

Supplementary information

Supplementary Figures

Supplementary Fig. S1. Gene map of (A) *Convallaria keiskei* and (B) *Liriope spicata*. Genes lying outside of the outer layer circle are transcribed in a counterclockwise direction, whereas genes inside this circle are transcribed in a clockwise direction. The colored bars indicate known protein-coding genes, tRNA genes, and rRNA genes. The dashed darker gray area in the inner circle denotes GC content, while the lighter gray area indicates genome AT content. LSC, large-single-copy; SSC, small-single-copy; IR, inverted repeat.

Supplementary Fig. S2. Sequence alignment of 16 chloroplast genomes in the Asparagales family performed using the mVISTA program with *Convallaria keiskei* as a reference. The top gray arrow shows genes in order (Transcriptional direction) and the position of each gene. A 70% cut-off was used for the plots. The Y-scale represents the percent identity between 50±100%. Red and blue areas indicate intergenic and genic regions, respectively. 1. *Convallaria keiskei*, 2. *Liriope spicata*, 3. *Maianthemum bicolor*, 4. *Nolina atopocarpa*, 5. *Polygonatum cyrtonema*, 6. *Asparagus officinalis*, 7. *Corydyline indivisa*, 8. *Esutrephus latifolius*, 9. *Aphyllanthes monspeliensis*, 10. *Mila biflora*, 11. *Anthericum ramosum*, 12. *Yucca brevifolia*, 13. *Agapanthus coddii*, 14. *Allium sativum*, 15. *Xanthorrhoeae preissii* and 16. *Aloe vera*.

Supplementary Fig. S3. Percentages of variable sites in protein-coding regions across the five Nolinoideae sub-family chloroplast genomes.
Supplementary Fig. S4: The schematic diagram represents the position of mitochondrial DNA in the *C. keiskei* cp genome.

Supplementary Fig. S5: Blast hit shows the sequence homology between mitochondrial DNA insert of *Convallaria keiskei* cp genome and *rpl10* of *Cocos nucifera* mitochondrial genome.

Supplementary Tables

Supplementary Table S1. Summary of chloroplast genome characteristics of *Convallaria keiskei* and *Liriope spicata*.

Supplementary Table S2. List of genes present in the cp genomes of *Convallaria keiskei* and *Liriope spicata*.

Supplementary Table S3. The list of pseudogenes, intron deletion gene, and gene inversion in the Asparagales order of plants.

Supplementary Table S4: List of primers used in this study.

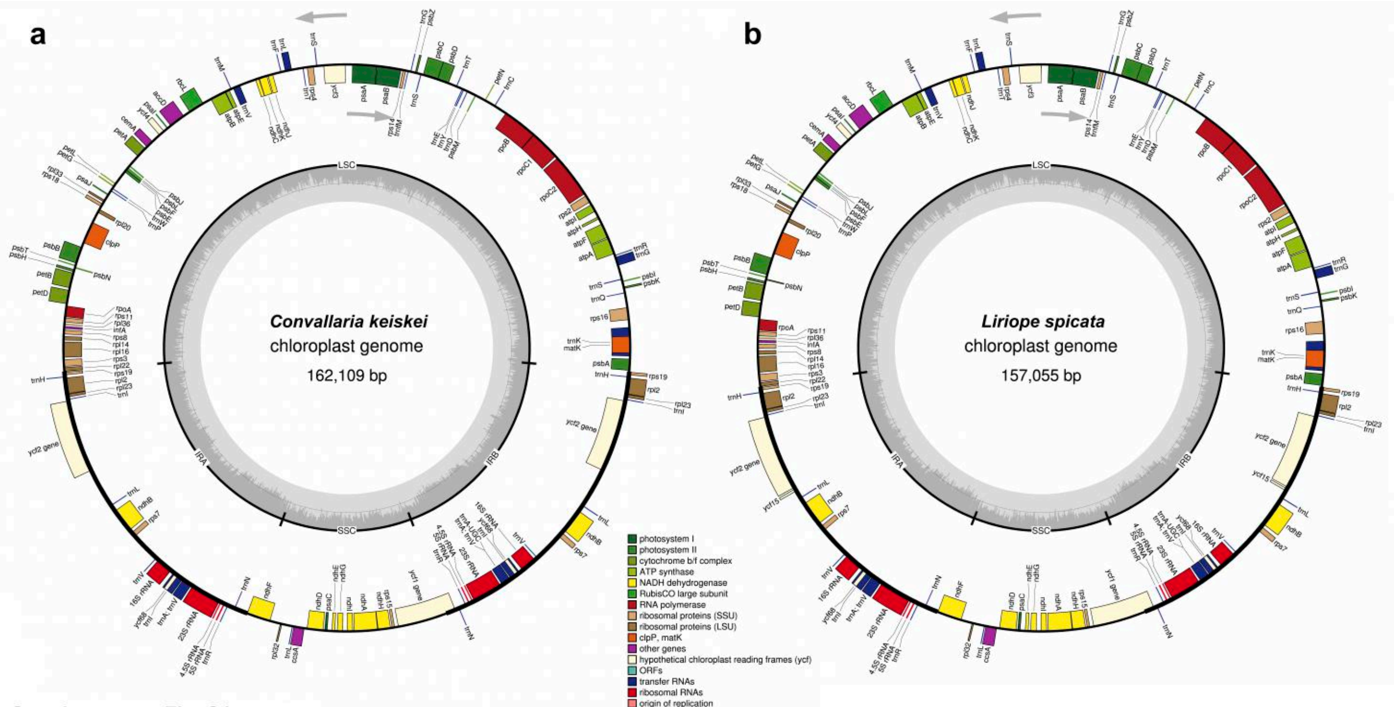

Supplementary Fig. S1

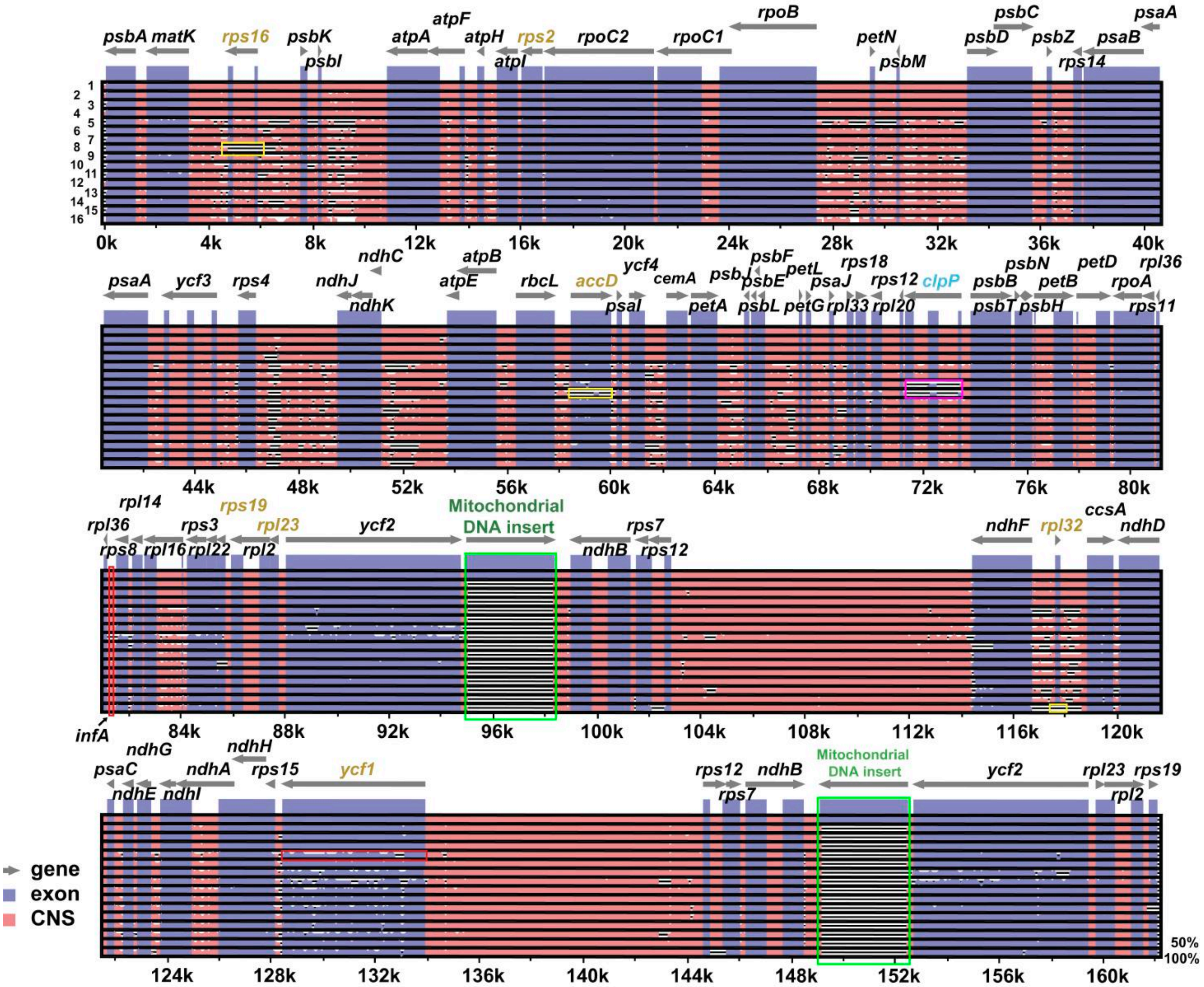

Supplementary Fig. S2

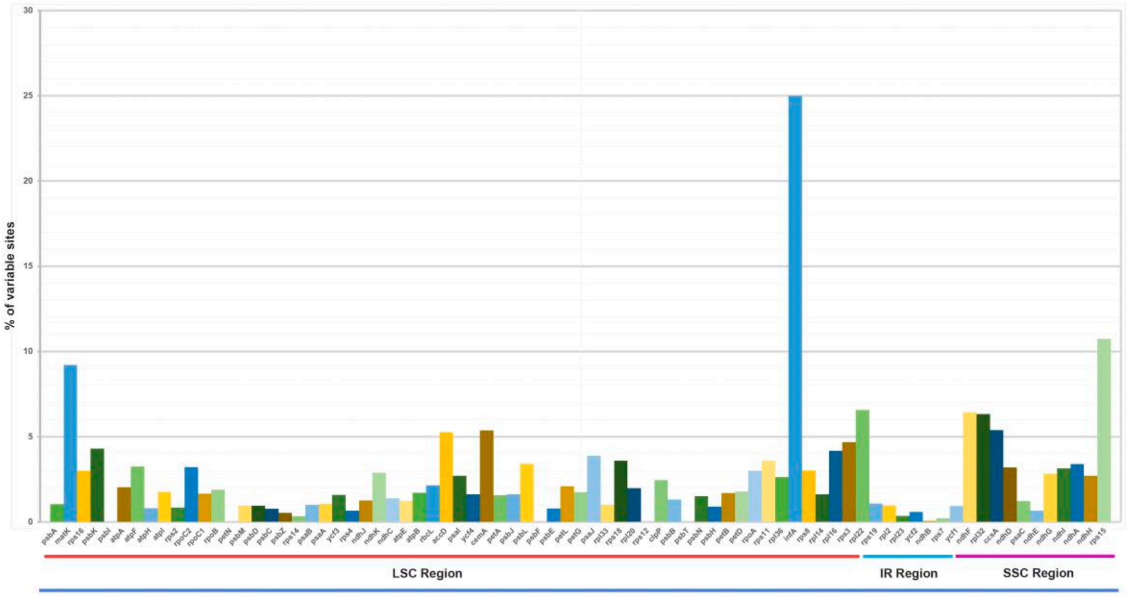

Supplementary Fig. S3 Protein-coding genes

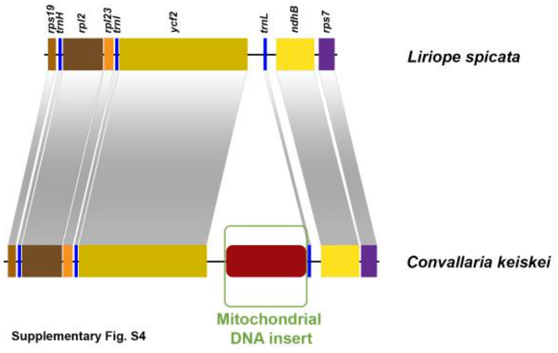

Supplementary Fig. S4

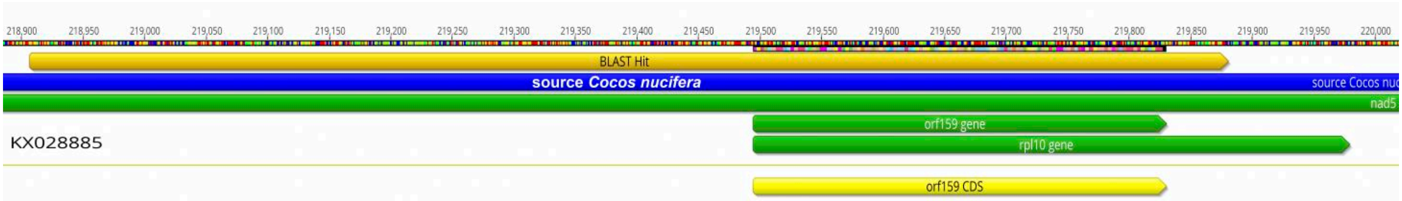

Supplementary Fig. S5

Supplementary Table S1. Summary of chloroplast genome characteristics of *Convallaria keiskei* and *Liriope spatia*

Genome features	*Convallaria keiskei*	*Liriope spatia*
Size (bp)	162,109	157,055
LSC length (bp)	85,344	85,374
SSC length (bp)	18,487	18,727
IR length (bp)	29,139	26,477
Total number of genes	131	131
Number of genes	112	112
Protein-coding genes	78+7	78+7
tRNA genes	30+8	30+8
rRNA genes	4+4	4+4
Number of genes duplicated in IR	19	19
GC content (overall %)	37.9	37.6
GC content - LSC (%)	35.7	35.6
GC content - SSC (%)	31.4	31.2
GC content - IR (%)	43.2	43.0

Supplementary Table S2. List of genes present in the cp genomes of *Convallaria keiskei* and *Liriope spatia*.

Category	Group of genes	Name of genes				
RNA genes	Ribosomal RNA genes	*rrn4.5*[a]	*rrn5*[a]	*rrn16*[a]	*rrn23*[a]	
	Transfer RNA genes	*trnA*-UGC[a, b]	*trnC*-GCA	*trnD*-GUC	*trnE*-UUC	*trnF*-GAA
		trnfM-CAU	*trnG*-GCC[b]	*trnG*-UCC	*trnH*-GUG[a]	*trnI*-CAU[a]
		trnI-GAU[a, b]	*trnK*-UUU	*trnL*-CAA[a]	*trnL*-UAA[b]	*trnL*-UAG
		trnM-CAU	*trnN*-GUU[a]	*trnP*-UGG	*trnQ*-UUG	*trnR*-ACG[a]
		trnR-UCU	*trnS*-GCU	*trnS*-GGA	*trnS*-UGA	*trnT*-GGU
		trnT-UGU	*trnV*-GAC[a]	*trnV*-UAC[b]	*trnW*-CCA	*trnY*-GUA
Photosynthetic genes	Subunits of photosystem I	*psaA*	*psaB*	*psaC*	*psaI*	*psaJ*
		ycf3[c]	*ycf4*			
	Subunits of photosystem II	*psbA*	*psbB*	*psbC*	*psbD*	*psbE*
		psbF	*psbH*	*psbI*	*psbJ*	*psbK*
		psbL	*psbM*	*psbN*	*psbT*	*psbZ*
	Subunits of cytochrome	*petA*	*petB*[b]	*petD*[b]	*petG*	*petL*
		petN				
	Subunits of ATP synthase	*atpA*	*atpB*	*atpE*	*atpF*[b]	*atpH*
		atpI				
	The large subunit of Rubisco	*rbcL*				
	Subunits of NADH dehydrogenase	*ndhA*[b]	*ndhB*[a, b]	*ndhC*	*ndhD*	*ndhE*
		ndhF	*ndhG*	*ndhH*	*ndhI*	*ndhJ*
		ndhK				
	ATP-dependent protease subunit P	*clpP*[c]				
	Chloroplast envelope membrane protein	*cemA*				
Transcription and translation-related genes	Small subunit of the ribosome	*rps2*	*rps3*	*rps4*	*rps7*[a]	*rps8*
		rps11	*rps12*[a, c, d]	*rps14*	*rps15*	*rps16*[e]
		rps18	*rps19*			
	Large subunit of ribosome	*rpl2*[a]	*rpl14*	*rpl16*[b]	*rpl20*	*rpl22*
		rpl23	*rpl32*	*rpl33*	*rpl36*	
	DNA-dependent RNA polymerase	*rpoA*	*rpoB*	*rpoC1*[b]	*rpoC2*	
	Translational initiation factor	*infA*[e]				

Other genes	Maturase	*matK*				
	Subunit of acetyl-CoA	*accD*				
	C-type cytochrome synthesis gene	*ccsA*				
	Component of TIC complex	*ycf1*[a, f]				
Genes of unknown function	Hypothetical proteins	*ycf2*[a],				

[a] - Two gene copies in IRs; [b] - Gene containing a single intron; [c] - Gene containing two introns; [d] - Gene divided into two independent transcription units; [e] – Pseudogene; [f] – Two copies – One intact gene and another is a pseudogene.

Supplementary Table S3. List of deleted genes in Asparagales

Sl. No.	Gene Name	Species Name	Sub-Family Name	GenBank Accession number
1.	*rpl32*	*Aloe vera*	Asphodeloideae	NC_035506
2.	*rpl23*	*Eustrephus latifolius*	Lomandroideae	NC_025305
3.	*rps19* - IRa	*Polygonatum cyrtonema*	Nolinoideae	NC_028429
4.	*rps19* - Both	*Hesperaloe parviflora*	Agavoideae	NC_032703
5.	*rps19* - Both	*Schoenolirion croceum*	Agavoideae	NC_032710
6.	*rps19* - Both	*Hosta ventricosa*	Agavoideae	NC_032706
7.	*rps19* - Both	*Hesperocallis undulata*	Agavoideae	NC_032704
8.	*rps19* - Both	*Hesperoyucca whipplei*	Agavoideae	NC_032705
9.	*rps19* - Both	*Chlorogalum pomeridianum*	Agavoideae	NC_032701
10.	*rps19* - Both	*Camassia scilloides*	Agavoideae	NC_032700
11.	*rps19* - Both	*Anthericum ramosum*	Agavoideae	NC_035972
12.	*rps16*	*Eustrephus latifolius*	Lomandroideae	NC_025305
13.	*rps16*	*Cordyline indivisa*	Lomandroideae	NC_035998
14.	*rps16*	*Allium sativum*	Allioideae	NC_031829
15.	*rps16*	*Hosta ventricosa*	Agavoideae	NC_032706
16.	*rps16*	*Beschorneria septentrionalis*	Agavoideae	NC_032699
17.	*rps2*	*Allium sativum*	Allioideae	NC_031829
18.	*rbcL*, *atpB*, *atpE* inversion	*Cordyline indivisa*	Lomandroideae	NC_035998
19.	*accD*	*Eustrephus latifolius*	Lomandroideae	NC_025305
20.	*clpP*	*Eustrephus latifolius*	Lomandroideae	NC_025305
21.	*clpP*	*Cordyline indivisa*	Lomandroideae	NC_035998
22.	*infA*	*Eustrephus latifolius*	Lomandroideae	NC_025305
23.	*infA*	*Allium sativum*	Allioideae	NC_031829
24.	*infA*	*Anthericum ramosum*	Agavoideae	NC_035972
25.	*infA*	*Aphyllanthes monspeliensis*	Aphyllanthoideae	NC_035968
26.	*infA*	*Aloe vera*	Asphodeloideae	NC_035506
27.	*infA*	*Milla biflora*	Brodiaeoideae	NC_036000
28.	*infA*	*Camassia scilloides*	Agavoideae	NC_032700
29.	*infA*	*Yucca brevifolia*	Agavoideae	NC_032711
30.	*infA*	*Hesperocallis undulata*	Agavoideae	NC_032704
31.	*infA*	*Hosta ventricosa*	Agavoideae	NC_032706
32.	*infA*	*Nolina atopocarpa*	Nolinoideae	NC_032708
33.	*infA*	*Convallaria majalis*	Nolinoideae	In this study
34.	*infA*	*Liriope spatia*	Nolinoideae	In this study
35.	*ycf1*	*Polygonatum cyrtonema*	Nolinoideae	NC_028429

Supplementary Table S3. The list of pseudogenes, intron deletion gene, and gene inversion in the Asparagales order of plants.

**Sub-Family Name**	**Species Name**	*accD*	*clpP*[#]	*infA*	*rbcL-atpE**	*rpl23*	*rpl32*	*rps2*	*rps16*	*rps19*[@]	*ycf1*[$]
Agavoideae	*Anthericum ramosum*										
	Beschorneria septentrionalis										
	Camassia scilloides										
	Chlorogalum pomeridianum										
	Hesperaloe parviflora										
	Hesperocallis undulata										
	Hesperoyucca whipplei										
	Hosta ventricosa										
	Schoenolirion croceum										
	Yucca brevifolia										
Allioideae	*Allium sativum*										
Aphyllanthoideae	*Aphyllanthes monspeliensis*										
Asparagoideae	*Asparagus officinalis*										
Asphodeloideae	*Aloe vera*										
Brodiaeoideae	*Milla biflora*										
Lomandroideae	*Cordyline indivisa*										
	Eustrephus latifolius										
Nolinoideae	*Convallaria keiskei*[1]										
	Liriope spatia[2]										
	Mianthemum bicolor										
	Nolina atopocarpa										
	Polygonatum cyrtonema										

[#] Two introns of *clpP* gene were lost in the cp genome.

* The protein-coding genes *rbcL*, *atpB* and *atpE* inverted in the LSC region.

[@] *rps19* – Two copies of *rps19* genes were deleted in IR regions of above mentioned species except for *Polygonatum crytonema*.

[$] *ycf1* – Two copies of ycf1 were pseudogenes in *Polygonatum crytonema*.

[1, 2] These plants were used in the present study.

Supplementary Table S4. List of primers used in this study

Primer name	Primer sequence position (bp)	Primer location region	Primer sequence (5' - 3' direction)	Product size (bp)
1F	94,245 – 94,269	*ycf2*	GGAACCATGCAGTACCAGACACGAG	4,910
1R	99,131 – 99,154	*ndhB*	ACCAAGAAATAACCCCTCACGTGCG	
2F	87,259 – 87,283	*rpl2*	TGCCTTGCGGTAATGATTCCTCTGG	8,943
2R	96,178 – 96,201	Mitochondrial DNA region	AGAACCCCATTTCGACCAAGTTCCG	
3F	96,238 – 96,262	Mitochondrial DNA region	GTTACTGGTCGAGCCACTGGAATGG	8,867
3R	105,080 – 105,104	*rrn16*	AAGGGCAGGTTCTTACGCGTTACTC	
4F	87,259 – 87,283	*rpl2*	TGCCTTGCGGTAATGATTCCTCTGG	17,846
4R	105,080 – 105,104	*rrn16*	AAGGGCAGGTTCTTACGCGTTACTC	
